# Prenatal Delta-9-Tetrahydrocannabinol Exposure Induces Transcriptional Alterations in Dopaminergic System with Associated Electrophysiological Dysregulation in the Prefrontal Cortex of Adolescent Rats

**DOI:** 10.3390/cells14120904

**Published:** 2025-06-14

**Authors:** Martina Di Bartolomeo, Sonia Aroni, Marcello Serra, Valeria Serra, Francesca Martella, Federica Gilardini, Miriam Melis, Claudio D’Addario

**Affiliations:** 1 Department of Bioscience and Technology for Food, Agriculture and Environment, University of Teramo, 64100 Teramo, Italy; mdibartolomeo@unite.it (M.D.B.); fmartella@unite.it (F.M.); federica.gilardini@unicam.it (F.G.); 2 Department of Biomedical Sciences, Division of Neuroscience and Clinical Pharmacology, University of Cagliari, 09100 Cagliari, Italy; sonia.aroni@unica.it (S.A.); marcelloserra@unica.it (M.S.); valeria.serra@unica.it (V.S.); 3 Department of Clinical Neuroscience, Karolinska Institutet, 17177 Stockholm, Sweden

**Keywords:** dopamine D2 receptor, dopamine D1 receptor, DNA methylation, electrophysiology, miRNAs, Netrin-1, sex, THC

## Abstract

Prenatal cannabis exposure (PCE) has been associated with altered prefrontal cortex (PFC) activity and connectivity in adulthood, potentially increasing the risk of psychopathology later in life. This risk is thought to involve a complex interplay between the endocannabinoid and dopaminergic systems. We investigated the transcriptional regulation of genes associated with these systems in an animal model of PCE during adolescence, focusing on DNA methylation and specific microRNAs (miRNAs). Our study revealed increased mRNA levels of dopamine D1 and D2 receptors (*Drd1* and *Drd2*) in the PFC, with a notable effect on *Drd2* in male offspring. Notably, we observed a consistent reduction in *Drd2* DNA methylation levels in PCE male rats. Both *Drd1* and *Drd2* expressions were regulated by selective miRNAs. Accordingly, we found changes in the excitability of PFC pyramidal neurons in male adolescent PCE offspring, along with alterations in the Netrin-1/DCC guidance cue system. Our findings highlight PCE-induced modifications of the PFC dopaminergic system while maintaining stable gene expression of the endocannabinoid system in male offspring. Changes in this complex interaction during sensitive developmental periods like adolescence might lead to sex-dependent divergent behavioral outcomes induced by PCE.

## 1. Introduction

*Cannabis sativa* is the most cultivated, trafficked, and abused drug worldwide. Survey data from 2015 to 2020 indicates that 15.4% of European citizens between the ages of 15 and 35 years reported using cannabis in the preceding year [1]. Notably, cannabis is the most commonly used drug among pregnant women [2,3], with up to 50% of consumers continuing its use during pregnancy [4,5,6,7]. Moreover, the prevalence and frequency of prenatal cannabis use are constantly increasing, driven by the legalization and commercialization in several countries [8,9,10]. The expansion of the retail cannabis marketplace and the introduction of novel cannabis products [11] have contributed to the common misperception of cannabis as a safe substance [12,13,14,15]. However, the average content of Delta-9-tetrahydrocannabinol (THC), the main psychoactive component of the *Cannabis sativa* plant, in cannabis preparations has increased dramatically over the last years, suggesting that cannabis is becoming increasingly harmful [16]. Notably, THC can cross the placenta [17] and accumulates into the maternal breast milk [18,19]. Thus, prenatal cannabis exposure (PCE) may negatively affect neurodevelopment during early and sensitive stages of brain growth, such as the pre-/perinatal stages, with a potential impact on neurological, behavioral, and executive functions [20,21].

Studies performed on large prospective longitudinal cohorts [22,23,24,25,26,27,28,29,30,31,32,33,34] have highlighted the detrimental effects of PCE on cognitive functions. PCE induces deficits in memory, verbal reasoning, concentration, and attention alongside heightened hyperactivity, impulsivity, and aggression [35,36,37]. PCE is also predictive of poorer academic achievement, reading, and spelling scores [28] as well as altered executive function and visuospatial working memory processing [21,25,26]. However, as suggested by Torres and colleagues [38], there is no complete consensus on the impact of PCE on cognitive functioning in the literature, emphasizing the necessity for a comprehensive investigation into the underlying mechanisms. Among these latter, special attention should be paid to the potential neurodevelopmental deficits induced by PCE in the prefrontal cortex (PFC), investigating its long-term effects [39]. In this context, it should be recalled that cognitive deficits are common features of neuropsychiatric disorders, including schizophrenia (SCZ) [40]. Dysfunctions in PFC networks are commonly observed in psychosis [41,42,43,44,45,46] and, when occurring during sensitive stages of neurodevelopment, they may represent a predictive marker of subsequent psychopathology [47,48]. In particular, cannabis exposure during critical PFC developmental periods has been implicated in the etiology of various neuropsychiatric disorders [49,50,51]. Interestingly, longitudinal human studies highlight how children prenatally exposed to cannabis exhibit increased vulnerability to psychosis symptoms [52,53], as well as in utero cannabis exposure is associated with child psychotic-like experiences [54]. Moreover, in adolescence, PCE is associated with aggressive rule-breaking behaviors [27,32,52].

Given the dopaminergic hypothesis of psychosis, which postulates that dopamine abnormalities in the mesolimbic and prefrontal brain regions are the basis of SCZ [55], it is particularly relevant to consider how PCE induces alterations in gene expression and the function of several neurotransmitters and receptors, among which dopamine stands out [56,57]. In animal models, PCE effects on dopamine function during early brain development translate into cognitive function alterations and sensitivity to substance use disorders [58,59,60,61]. Moreover, PCE induces alterations in dopamine receptor function [62,63] with the hyper-responsiveness of the mesocorticolimbic dopamine system [63,64,65,66]. Furthermore, there is a functional interaction between the dopaminergic and the endocannabinoid system (ECS), the molecular target of exogenous cannabinoids, in terms of receptor co-expression [67] and signal transduction convergence [68,69]. The ECS is a key homeostatic system found throughout the body, present in both the central and peripheral nervous systems as well as in other peripheral tissues [70]. It consists of the primary cannabinoid receptors type 1 (CB1) and type 2 (CB2), the endogenous ligands anandamide “AEA” and 2-arachidonoylglycerol “2-AG”, and the enzymes responsible for synthesis and degradation [70]. Among the plethora of processes in which it is involved, the ECS has attracted attention for its neuromodulatory activity in the central nervous system, influencing emotional processing, motivational behavior, and cognitive function [71,72,73]. Of note, ECS components are critically involved in fetal neurodevelopment, particularly in processes such as neuronal cell proliferation and differentiation and synaptic plasticity [57,74,75]. PCE may disrupt the temporal regulation of the cannabinoid CB1 receptor gene (Cnr1), which is expressed and functional from early embryonic development [76]. Consequently, the overstimulation of Cnr1 could interfere with neurodevelopmental processes, leading to alterations in ECS signaling. Altogether, this can lead to aberrant neurodevelopment and an increased risk for substance use disorders and help explain how pre-/perinatal cannabis exposure increases the risk of these conditions during life [77,78,79]. Environmental factors, including cannabis exposure, may contribute to the development of psychotic-like symptoms through modifications of the epigenome [80,81]. Epigenetics is a crucial player in neuronal development, differentiation, and communication as well as in synaptic plasticity [82]. Of note, epigenetic mechanisms, including DNA methylation and miRNAs, hold particular promise for understanding complex disorders such as neuropsychiatric ones, as they reveal molecular links between environmental factors and gene regulation. Specifically, epigenetic mechanisms act as a critical interface connecting environmental influences to genomic activity [83]. This dynamic is especially relevant in neurofunctional disorders like SCZ which often involve significant environmental contributions alongside genetic predisposition [84]. Studies on cannabinoid animal models showed how PCE can impact DNA methylation in the genomes of exposed offspring [85,86,87,88,89]. Likewise, cannabis can affect the sperm methylome of cannabis male users, impacting genes involved in neuronal development signaling [90,91]. In addition, cannabinoid exposure can also induce alterations in miRNAs profiles in the brain, peripheral blood, and gut [92,93,94,95]. Of note, PCE affects miRNAs expression in the fetal cerebrospinal fluid of rhesus macaques [96] as well as in adult rat ovaries [97].

The PFC regulates advanced cognitive functions, including planning, working memory, decision-making, and the modulation of social behaviors [98]. Although PFC neurons are generated prenatally, their differentiation and the formation of synaptic connections in humans extend into the third decade of life [99]. The mesocorticolimbic dopamine system is essential for regulating PFC functions [100], and this process is orchestrated by a complex interplay of molecular signals [41,101,102]. Notably, the Netrin1/DCC (Deleted in Colorectal Cancer) signaling pathway has emerged as a critical regulator of axonal growth and neural connectivity during adolescence [103]. This extended maturation period allows for significant refinement of neural circuitry based on individual experiences; however, it also renders the PFC particularly susceptible to disruptions [47]. PCE critically affects fetal cortical circuitry transmission [21,104]. However, to date, no evidence shows that, among PCE’s detrimental effects, Netrin-1/DCC signaling could be disrupted during adolescence.

On this basis, the present study aims to investigate if and how PCE alters the development of rat PFC in adolescence. For this purpose, we investigated if PCE impaired the neurodevelopment of the PFC by examining the transcriptional regulation of the ECS and dopaminergic system genes, the excitability of pyramidal neurons, as well as the Netrin-1/DCC guidance cue system in both sexes. PCE induced changes in the transcriptional regulation of genes within the dopaminergic system but not the endocannabinoid system. Notably, these transcriptional changes were specific to the dopamine D2 receptor gene (*Drd2*) and accompanied by increased excitability of pyramidal neurons in the PFC of male adolescent rats along with modifications of the Netrin-1/DCC signaling.

## 2. Materials and Methods

### 2.1. Subjects and Treatments

#### 2.1.1. Drugs

THC resin was purchased from THC PHARM GmbH (Frankfurt, Germany), dissolved in ethanol at a 20% final concentration, and then sonicated for 30 min. THC was emulsified in 1–2% Tween^®^ 80 (Sigma-Aldrich, St. Louis, MO, USA) and then dissolved in sterile saline (0.9% NaCl).

#### 2.1.2. Animals and Treatments

We took advantage of a well-established PCE rat model [60,63,65,66,105,106]. Primiparous female Sprague Dawley rats (Envigo) were used as mothers and single-housed during pregnancy. Delta-9-tetrahydrocannabinol (THC) or vehicle was administered (2 mg/kg/mL, s.c. once daily) from gestational day (GD) 5 to GD20. We administered THC or its vehicle during the 2nd and 4th hour of light in the light/dark cycle. The first day of pregnancy was identified based on the collection of the vaginal plug that defined GD1. THC dose was chosen due to its lack of behavioral responses or cannabinoid tolerance after repeated administration [107]. This concentration (5%) does not have any substantial impact on maternal and non-maternal behavior, as well as offspring body weight [65]. Moreover, it is equivalent in rodent plasma concentrations (8.6–12.4 ng/mL) to human recreational cannabis smokers (from a 7% THC) 0–22 h post-inhalation (13–63 ng/mL) [108,109]. As we previously described in this PCE model, and according to the literature [110], we found no differences in litter size, male/female ratio, or body weight at preadolescence (PND25-26) among vehicle and PCE offspring [65]. As previous studies did not measure birth weight, we did not evaluate this parameter [60,63,64,65,66,89,105,106,111,112]. However, a previous study that used a similar prenatal THC dose regimen highlighted a decreased birth weight in PCE offspring that was reverted at postnatal day (PND) 21 [110], thus suggesting that catch-up growth might explain the normal body weight found at PND25-26 in PCE offspring. The offspring were weaned at postnatal day (PND) 21 and were housed in a climate-controlled animal room (21 ± 1 °C; 60% humidity) under a normal 12 h light–dark cycle (lights on at 7:00 a.m.) with water and food available ad libitum until the experimental day (PND50-55). To control for litter effects, we did not use more than two offspring from each litter for the same experiment.

All procedures were performed in accordance with the European legislation (EU Directive, 2010/63) and were approved by the Animal Ethics Committees of the University of Cagliari and by the Italian Ministry of Health (auth. n. 636/2022-PR). All possible efforts were made to minimize animal pain and discomfort and to reduce the number of experimental subjects.

### 2.2. Molecular Biology Analysis

Molecular analyses were performed on RNA and DNA isolated from the dissected PFC of 15 control and 14 PCE rats.

#### 2.2.1. Gene Expression Analysis by Quantitative Real-Time Polymerase Chain Reaction (RT-qPCR)

Total RNA was extracted from dissected rat PFC tissue using Qiazol^®^ Reagent (Qiagen, Hilden, DE, Germany) following the manufacturer’s instructions. The concentration of each purified RNA sample was measured using a NanoDrop 2000c UV–Vis Spectrophotometer (Thermo Fisher Scientific, Waltham, MA, USA). The A260/A280 absorbance ratio was used to evaluate protein contamination, with values between 1.8 and 2.1 considered acceptable. An amount of 1 µg of total RNA was converted into cDNA by a SensiFAST^TM^ cDNA Synthesis kit (Bioline Reagents, London, UK). Real-time quantitative polymerase chain reaction (RT-qPCR) was performed using a SensiFAST^TM^ SYBR^®^ Lo-ROX Kit (Bioline Reagents, London, UK) on a DNA Engine Opticon 2 Continuous Fluorescence Detection System (MJ Research). To accurately quantify the initial target in each PCR reaction, the amplification plot was analyzed, and the point of the early log phase of product accumulation was identified by setting a fluorescence threshold above the background signal. This point, known as the threshold cycle number (Ct), was determined as previously described [113,114,115]. Differences in threshold cycle values were used to quantify the relative amounts of PCR targets in each sample. Following PCR, a dissociation (melting) curve was generated from 60 to 95 °C [116] to assess the specificity of the amplified products. The relative expression levels of the amplicons were calculated using the Delta–Delta Ct (ΔΔCt) method and transformed to 2^−ΔΔCt^ for statistical analysis [117]. All data were normalized using three endogenous reference genes: glyceraldehyde-3-phosphate dehydrogenase (GAPDH), beta-actin (β-ACT), and 18s ribosomal RNA (18S). The primer sequences used for amplification are listed in Table 1.

#### 2.2.2. DNA Methylation Analysis by Pyrosequencing

After extraction using Qiazol^®^ Reagent (Qiagen, Hilden), DNA concentrations were determined by measuring absorbance at 260 nm, while sample purity was assessed using the absorbance ratio at 260 nm to 280 nm (A260/A280 = 1.8). Each purified DNA sample was subjected to bisulfite modification using the EZ DNA Methylation-Gold™ Kit (Zymo Research, Orange, CA, USA) according to the manufacturer’s instructions. *Drd1* and *Drd2* (coding for the dopaminergic receptor D1 and D2, respectively, see Supplementary Figure S1 for gene details) DNA methylation was subsequently evaluated using a pyrosequencing assay purchased from Qiagen.

Bisulfite-treated DNA was first amplified by a PyroMark PCR Kit (Qiagen, Hilden, DE) with a biotin-labeled primer according to the manufacturer’s recommendations [114,115,118]. PCR conditions were as follows: 95 °C for 15 min, followed by 45 cycles of 94 °C for 30 s, 56 °C for 30 s, 72 °C for 30 s, and, finally, 72 °C for 10 min. The specificity of PCR products was then verified by electrophoresis.

The sequencing was performed on a PyroMark Q48 Autoprep using PyroMark Q48 Advanced Reagents (Qiagen, Hilden, DE), following the manufacturer’s recommendations. Primers for the PCR amplification and sequencing of rat *Drd1* were designed using PyroMark Assay Design Software version 2.0 (Qiagen, Hilden, Germany) to target four CpG sites within the gene’s regulatory region. A specific PyroMark CpG assay (Qiagen, Hilden, Germany) was instead employed to analyze six CpG sites in the rat *Drd2* gene regulatory region. The DNA methylation level was analyzed through the PyroMark Q48 Autoprep 2.4.2 software, which calculates the methylation percentage mC/(mC + C) (mC = methylated cytosine, C = unmethylated cytosine) for each CpG site, allowing quantitative comparisons. Quantitative methylation results were expressed both as a percentage of every single CpG site and the average of the methylation percentage of all the CpG sites under study. Details of the sequences analyzed, along with the primers and assays used, are provided in Table 2.

#### 2.2.3. miRNAs Expression Analysis by Quantitative Real-Time Polymerase Chain Reaction (RT-qPCR)

Total RNA was extracted from dissected rat PFC tissue using Qiazol^®^ Reagent (Qiagen, Hilden, DE) following the manufacturer’s instructions. The concentration of each purified RNA sample was measured using a NanoDrop 2000c UV–Vis Spectro-photometer (Thermo Fisher Scientific, Waltham, MA, USA). The A260/A280 absorbance ratio was used to evaluate protein contamination, with values between 1.8 and 2.1 considered acceptable.

A miRCURY LNA RT Kit (Qiagen, Hilden, DE) was used to perform miRNA polyadenylation and reverse transcription in a single reaction step. Real-time quantitative polymerase chain reaction (RT-qPCR) was performed using SensiFAST^TM^ SYBR^®^ Lo-ROX Kit (Bioline Reagents, London, GB) on a QIAquant System (Qiagen, Hilden, DE).

To accurately quantify the initial target in each PCR reaction, the amplification plot was analyzed, and the point of the early log phase of product accumulation was identified by setting a fluorescence threshold above the background signal. This point, known as the threshold cycle number (Ct), was determined as previously described [113,114,115]. Following PCR, a dissociation (melting) curve was generated from 60 to 95 °C [116] to assess the specificity of the amplified products. The relative expression levels of the amplicons were calculated using the Delta–Delta Ct (ΔΔCt) method and transformed to 2^−ΔΔCt^ for statistical analysis [117]. All data were normalized to the U6 small nuclear RNA (miRCURY LNA miRNA PCR Assay YP02119464, Qiagen, Hilden, DE).

We investigated the expression of selected miRNAs targeting *Drd1* and *Drd2* and the PCR primer assays were the following: miR-30b-5p miRCURY LNA miRNA PCR Assay YP00204765; miR-17p-5p miRCURY LNA miRNA PCR Assay YP02119304; and miR-9-5p miRCURY LNA miRNA PCR Assay YP00204513 (Qiagen, Hilden, DE).

### 2.3. Electrophysiology

#### Electrophysiological Recordings

We prepared coronal prefrontal cortex (PFC) slices (300 μm) from PND50-55 offspring. Rats were anesthetized with isoflurane until the loss of the righting reflex, then were transcardially perfused with an ice-cold sucrose-based solution saturated with 95% O_2_/5% CO_2_, containing in mmol/L: 87 NaCl, 75 sucrose, 25 glucose, 5 KCl, 21 MgCl_2_, 0.5 CaCl_2_, and 1.25 NaH_2_PO_4_ [119,120]. The brain was rapidly removed, and PFC slices were obtained using the same sucrose-based solution kept at 4 °C with a vibratome (Leica VT 1000S).

Immediately after cutting, slices were stored for 1 h at 32 °C in an artificial cerebrospinal fluid (aCSF) at 304–306 mOsm and contained in mmol/L:130 NaCl, 11 glucose, 2.5 KCl, 1.2 MgCl_2_, 2.4 CaCl_2_, 23 NaHCO_3_, 1.2 NaH_2_PO_4_, and were equilibrated with 95% O_2_/5% CO_2_. Slices were then stored in aCSF at room temperature until recording.

Cells were visualized with an upright microscope with infrared illumination (Axioskop FS 2 plus; Zeiss, Oberkochen, Germany), and whole-cell patch-clamp recordings were made by using an Axopatch 200 B amplifier (Molecular Devices). Pyramidal neurons in the prelimbic portion of the PFC were identified by their pyramidal shape, the presence of a prominent apical dendrite, and the distance from the pial surface (layers V/VI). Current-clamp recordings were made with electrodes (resistance of 4–6 MΩ) filled with a solution containing the following (in mM): 144 KCl, 10 HEPES buffer, 3.45 BAPTA, 1 CaCl_2_, 2.5 Mg_2_ATP, and 0.25 Mg_2_GTP, pH 7.3–7.4, 283–285 mOsm. Current-clamp experiments were performed in the absence of any pharmacological blocker (regular aCSF). Experiments began only after series resistance had stabilized (typically 15–40 MΩ). Data were filtered at 2 kHz, digitized at 10 kHz, and collected online with acquisition software (pClamp 10.2, Molecular Devices). The membrane potential was held near −65 mV, and evoked firing was measured using depolarizing current steps (0.4 s) from 0 to 400 pA.

### 2.4. Immunohistochemistry

#### 2.4.1. Tissue Preparation

At PND 50–55, rats were deeply anesthetized with isoflurane and transcardially perfused with saline, followed by 4% paraformaldehyde in 0.1 M phosphate buffer (PB; pH = 7.4). Afterward, brains were removed, postfixed 2 h in the same solution at 4 °C, then rinsed three times in PB saline 1× (PBS) and preserved in the same solution at 4 °C. The next day, brains were coronally cut on a vibratome (VT1000S, Leica Biosystems) to yield sections (thickness, 40 μm) suited for immunohistochemistry (IHC) processing. For each rat, three coronal sections representative of the medial prefrontal cortex (mPFC) and nucleus accumbens (NAc), containing both the core and shell substructures, were collected based on stereotaxic coordinates ranging from 3.20 mm to 2.50 mm relative to bregma. These coordinates were referenced from the rat brain atlas by Paxinos and Watson [121].

#### 2.4.2. Reaction Protocol, Image Acquisition, and Density Analysis

For sections used for Netrin-1 IHC only, antigen retrieval was performed by placing sections in sodium citrate buffer (pH 6) for 25 min at 95 °C. Then, free-floating sections were rinsed in 0.1 M PB and blocked in a solution containing 10% normal goat serum (Vector, London, UK) and 0.5% Triton X-100 in 0.1 M PB at room temperature (2 h). Thereafter, sections were incubated at 4 °C with either the rabbit polyclonal primary antibody anti-tyrosine hydroxylase (TH, 1:1000, Merck, Darmstadt, Germany, #AB152, 48 h) or rabbit polyclonal primary antibody anti-Netrin-1 (1:600, Alomone Labs, Jerusalem, Israel, #ANR-121, 96 h), rinsed three times in 0.1 M PB, and then incubated with the secondary antibody, Atto^®^ 488-labeled goat anti-rabbit IgG (1:400, Merck, Germany, #18772) in 0.1 M PB at room temperature (3 h). Afterward, sections were incubated for 10 min in 4′,6-diamidino-2-phenylindole (DAPI; 1:10,000, Merck, Italy, D9542) to allow the visualization of cell nuclei, rinsed in PB 0.1 M, and mounted onto super-frost glass slides using Mowiol^®^ mounting medium. Images of a single wavelength (14-bit depth) were obtained with a ZEISS Axio Scan Z1 slide scanner (Zeiss, Oberkochen, Germany). Brain sections were captured at 20× magnification (Objective: Plan-Apochromat 20×/0.8 M27) to acquire the whole mPFC and NAc from both hemispheres. The ImageJ software v.1.54, National Institutes of Health, Bethesda, Maryland, USA) was used to measure the density of TH and Netrin-1 signals in both brain areas. Images were converted to 8-bit, background-adjusted, and the signal density was quantified in regions of interest representative of each brain area, with dimensions of 300 × 300 µm for TH and 400 × 400 µm for Netrin-1. Density analyses in the NAc reported the sum of measurements from both the core and shell subregions. Analyses were conducted blind to the treatment of each animal. No significant differences in the relative density of TH and Netrin-1 immunoreactivity were found among the three sections, therefore values from different antero-posterior levels were averaged.

### 2.5. Statistical Analysis

All results were expressed as the mean ± standard error of the mean (SEM). Statistical differences between the experimental groups were evaluated using GraphPad Prism^®^ 9 (Graph-Pad Software, San Diego, CA, USA). 

Different statistical approaches were employed based on the research questions and the existing literature. For molecular analyses (gene expression, DNA methylation, and miRNA expression), we first examined overall PCE effects in the combined sample, followed by sex-stratified analyses to increase sensitivity for detecting potentially small sex-dependent differences in molecular regulation. This approach was chosen because molecular alterations may manifest in distinct ways or with different magnitudes between sexes. For electrophysiological recordings, a factorial design (2-way RM ANOVA) was employed because these measurements generate continuous parametric data across multiple stimulation intensities, allowing for a direct comparison of treatment effects between sexes and the assessment of potential interaction effects. Similarly, immunohistochemical data were analyzed using 2-way ANOVA to directly assess the main effects of both PCE and sex, as well as their potential interaction, on protein expression levels in different brain regions. These region-specific analyses allowed us to evaluate whether PCE effects might be differentially expressed across neural circuits.

The nonparametric Mann–Whitney test was used to assess changes in gene and miRNAs expression levels first in the combined population and then in the sex-stratified one. DNA methylation level at each CpG site as well as in their average was analyzed using the Mann–Whitney test and Holm–Sidak correction was used for multiple comparisons, as suggested. Two-way RM ANOVA and multiple comparisons with Holm–Sidak were used to evaluate pyramidal neuron excitability. The nonparametric Mann–Whitney test was used for analyzing the intrinsic properties of pyramidal neurons. Two-way ANOVA followed by Tukey’s post-hoc test was used to analyze changes in TH and Netrin-1 immunoreactivity. All the correlations were performed by Spearman’s rank coefficient. *p*-values < 0.05 were considered to be statistically significant. 

While stratified analyses by sex were employed to detect potential sex-dependent differences, we acknowledge that this approach increases the risk of Type I error due to multiple comparisons. We did not apply additional corrections for multiple comparisons in our sex-stratified analyses, which represents a limitation of this statistical approach. Some of our sex-stratified analyses should be considered exploratory in nature, providing direction for future studies specifically designed to test sex-dependent effects with appropriate statistical power. For the electrophysiological data, we utilized a factorial design because previous studies found that PCE specifically modifies these parameters only in male offspring [65,105,111]. 

## 3. Results

### 3.1. PCE Alters Drd1 and Drd2 Gene Expression in Prefrontal Cortex of Adolescent Offspring

To detect potential PCE-induced alterations in gene expression that might vary by sex, we first analyzed the combined sample, followed by sex-stratified analyses.

No significant differences in ECS gene mRNA levels were observed between PCE and control (CNT) rats in the PFC. See Table 3 for statistical details.

When analyzed by sex, no significant differences were observed between PCE and control animals in either males or females (See Supplementary Table S1). 

**Figure 1:**
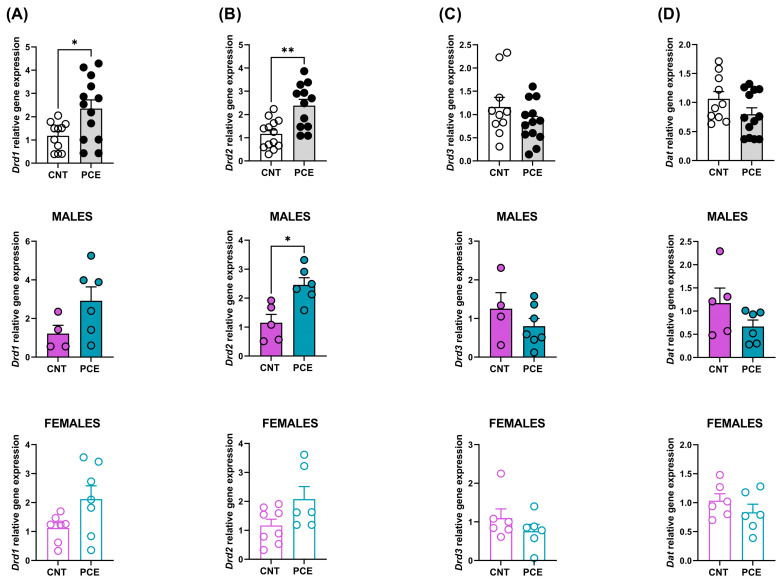
*Drd1* (**A**), *Drd2* (**B**), *Drd3* (**C**), and *Dat* (**D**) relative gene expression analyzed in the PFC of prenatal cannabis-exposed (PCE) and control (CNT) rats in the combined population and the population stratified according to sex. Gene expression data are reported as 2^−ΔΔCt^ values calculated by the Delta–Delta Ct (ΔΔCt) method versus CNT. Expression was normalized to GAPDH, β-actin, and 18S. Data are reported as mean ± SEM (n = 11–15 rats/group, combined population; n = 4–7 rats/group, male rats; n = 6–7 rats/group, female rats). Significant differences are indicated: Mann–Whitney Test ** *p* < 0.01, * *p* < 0.05 vs. CNT.

On the other hand, a significant increase was observed in *Drd1* mRNA levels in the PFC of PCE rats (2.34 ± 0.37) when compared to CNT (1.18 ± 0.18) (*p* = 0.0191, Figure 1A).

In sex-stratified analyses, no significant PCE effect was observed in either male or female animals (Figure 1A). This pattern of results—a significant effect in the combined sample without significant effects in either sex when analyzed separately—suggests that the PCE-induced increase in *Drd1* expression may be present in both sexes but with insufficient power to detect when the sample is split by sex.

Furthermore, a significant increase was also highlighted in *Drd2* mRNA levels in the PFC of PCE rats (2.37 ± 0.27) when compared to CNT (1.17 ± 0.17) (*p* = 0.0023, Figure 1B). Sex-stratified analysis revealed a significant increase in *Drd2* mRNA levels only in the PFC of male rats (CNT 1.15 ± 0.28, PCE 2.46 ± 0.25, *p* = 0.0173 Figure 1B). While PCE induced an increase in *Drd2* mRNA levels only in males, we cannot exclude the possibility of a smaller effect in females.

No significant differences were observed in *Drd3* and *Dat* mRNA levels between PCE and CNT animals (combined and sex-stratified population, Figure 1C,D).

### 3.2. PCE Alters Drd2 DNA Methylation in Prefrontal Cortex of Adolescent Offspring

Given the increased *Drd2* gene expression observed exclusively in male rats, we employed a similar analytical approach to examine DNA methylation patterns. 

**Figure 2:**
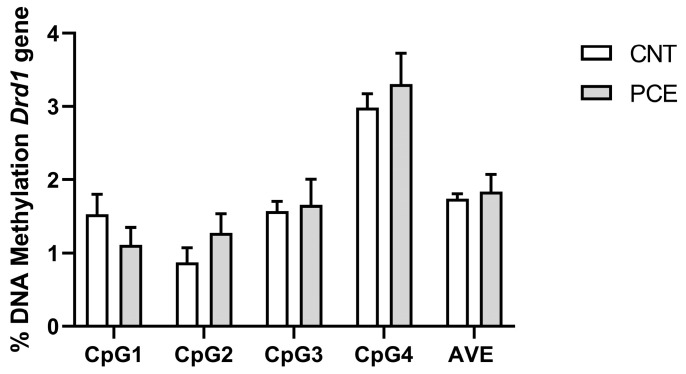
Comparison of DNA methylation status at rat *Drd1* gene in the PFC of prenatal cannabis-exposed (PCE) and control (CNT) rats. DNA methylation data are presented as the mean ± SEM of the methylation % values of individual CpG sites under study as well as of the average (AVE) of the four CpG sites (n = 15–14 rats/group).

No differences in *Drd1* DNA methylation levels were observed between PCE and CNT rats in the PFC (Figure 2).

**Figure 3:**
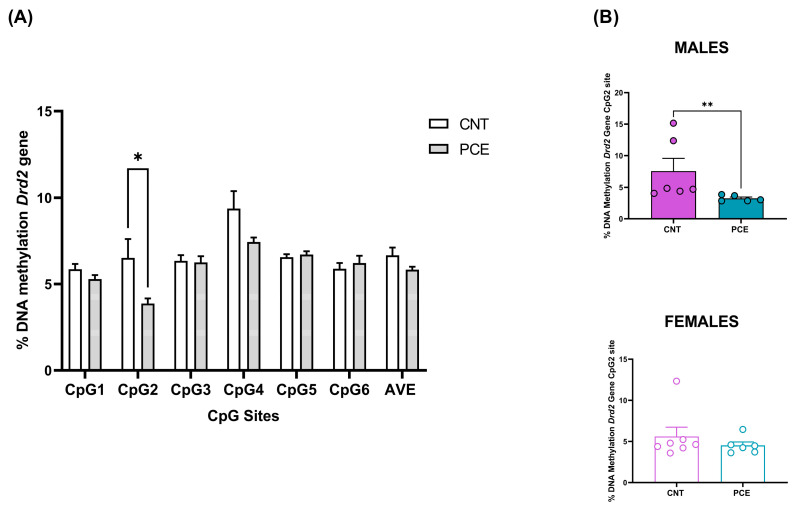
Comparison of DNA methylation status at rat *Drd2* gene in the PFC of prenatal cannabis-exposed (PCE) and control (CNT) rats in the combined study samples (**A**) and a comparison of *Drd2* DNA methylation status at the Cpg2 site in the PFC of prenatal cannabis-exposed (PCE) and control (CNT) rats stratified according to sex (**B**). DNA methylation data are presented as the mean ± SEM of the methylation % values of individual CpG sites under study as well as of the average (AVE) of the six CpG sites (n = 14 rats/group, combined population; n = 6–5 rats/group, male rats; n = 7–6 rats/group female rats). Significant differences are indicated: Mann–Whitney Test, Holm–Sidak corrected * *p* < 0.05 vs. CNT; ** *p* < 0.01 vs. CNT.

Conversely, consistent with the increase in mRNA levels, we observed a significant reduction in *Drd2* DNA methylation levels at the CpG2 site in the PFC of PCE rats (3.87 ± 0.29) when compared to the CNT (6.51 ± 1.09) (*p* = 0.031110, Figure 3A). Of note, consistent with our gene expression data, the sex-stratified analysis revealed a reduction in *Drd2* DNA methylation levels at CpG2 in male PCE rats (CNT 7.58 ± 1.99, PCE 3.25 ± 0.21, *p* = 0.0043 Figure 3B).

**Figure 4:**
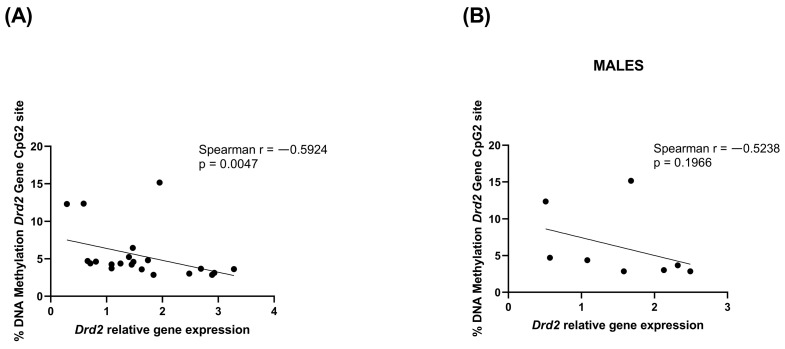
Correlation between *Drd2* relative gene expression and % change of *Drd2* DNA methylation at CpG2 site in rats PFC in the combined population (**A**) and in male rats only (**B**). Data were compared by Spearman’s rank correlation coefficient.

Moreover, a consistent and significant negative correlation between *Drd2* relative gene expression and DNA methylation percentage at the CpG2 site was observed when both male and female rats were considered (Spearman r = −0.5924, *p* = 0.0047) (Figure 4A). The correlation was not significant considering only male rats (Spearman r = −0.5238, *p* = 0.1966) (Figure 4B).

### 3.3. PCE Alters Selective miRNAs Expression, Targeting Drd1 and Drd2, in Prefrontal Cortex of Adolescent Offspring

To investigate further potential epigenetic mechanisms underlying the observed gene expression changes, we examined miRNAs expression using the same analytical approach. 

**Figure 5:**
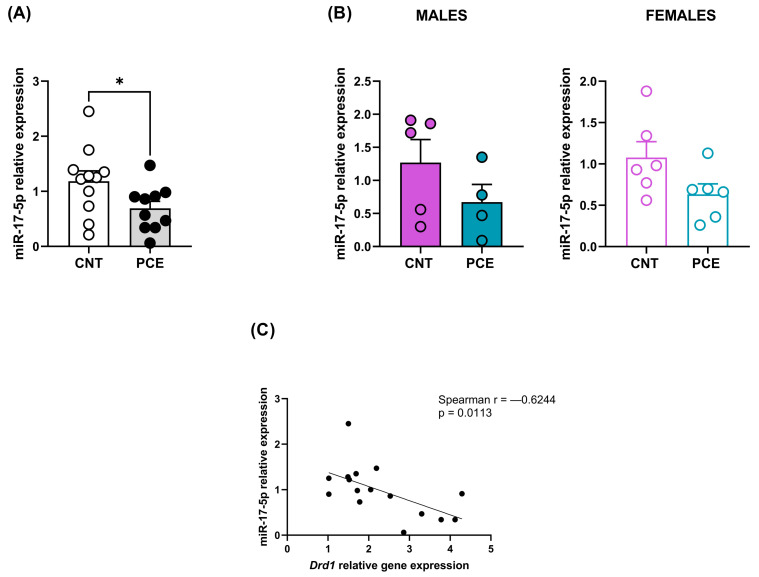
miR-17-5p expression levels (**A**) of the combined study samples (**A**) and male and female rats (**B**) and correlation between *Drd1* and miR-17-5p relative expression (**C**). miRNA expression data are reported as 2^−ΔΔCt^ values calculated by the Delta–Delta Ct (ΔΔCt) method versus the CNT. Expression was normalized to U6. Data are reported as the mean ± SEM (n = 11–10 rats/group, combined population; n = 5-4 rats/group, male rats; n = 6 rats/group, female rats). Significant differences are indicated: Mann–Whitney Test * *p* < 0.05 vs. CNT. For correlation, data were compared by Spearman’s rank correlation coefficient.

Consistent with the increase in *Drd1* mRNA levels, we highlighted a significant reduction in miR-17-5p expression levels in the PFC of PCE (0.69 ± 0.13) when compared to CNT rats (1.18 ± 0.19) (*p* = 0.0495, Figure 5A). The sex-stratified analysis revealed no differences in miR-17-5p levels in male and female rats (Figure 5B). The significant effect in the combined sample without significant differences in either sex when analyzed separately suggests that the PCE-induced reduction in miR-17-5p expression likely occurs in both sexes.

Of note, we observed a consistent and significant negative correlation between *Drd1* mRNA levels and miR-17-5p expression (Spearman r = −0.6244, *p* = 0.0113) (Figure 5C). No differences were observed for miR-30b-5p relative expression (Supplementary Figure S2).

**Figure 6:**
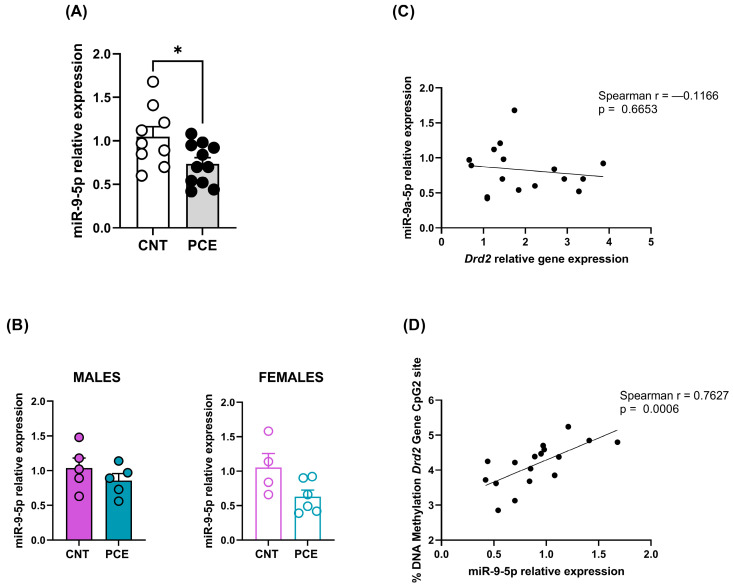
miR-9-5p expression levels in the PFC of the combined study samples (**A**) and male and female rats (**B**), correlation between *Drd2* and miR-9-5p relative expression (**C**) and between miR-9-5p relative expression and *Drd2* DNA methylation levels at CpG2 site (**D**). miRNA expression data are reported as 2^−ΔΔCt^ values calculated by the Delta–Delta Ct (ΔΔCt) method versus CNT. Expression was normalized to U6. Data are reported as the mean ± SEM (n = 9–11 rats/group, combined population; n = 5 rats/group, male rats; n = 4–6 rats/group, female rats). Significant differences are indicated: Mann–Whitney Test * *p* < 0.01 vs. CNT. For correlation, data were compared by Spearman’s rank correlation coefficient.

Interestingly, consistent with the increase in *Drd2* mRNA levels, we observed a significant reduction in miR-9-5p expression levels in the PFC of PCE (0.73 ± 0.07) when compared to CNT rats (1.05 ± 0.11) (*p* = 0.0461, Figure 6A). Sex-stratified analysis revealed no significant differences between male and female rats (Figure 6B). As with miR-17-5p, the significant reduction in miR-9-5p in the combined sample without significant effects in either sex individually suggests that PCE may affect miR-9-5p expression in both sexes, but with insufficient power to detect when the sample is split by sex. 

Similarly, the correlation between *Drd2* and miR-9-5p expression levels was not significant (Figure 6C). Conversely, a significant correlation was highlighted between miR-9-5p expression levels and *Drd2* DNA methylation levels at the CpG2 site (Spearman r = 0.7627, *p* = 0.0006 Figure 6D).

### 3.4. PCE Alters the Excitability of Medial Prefrontal Cortex Pyramidal Neurons in Adolescent Offspring

**Figure 7:**
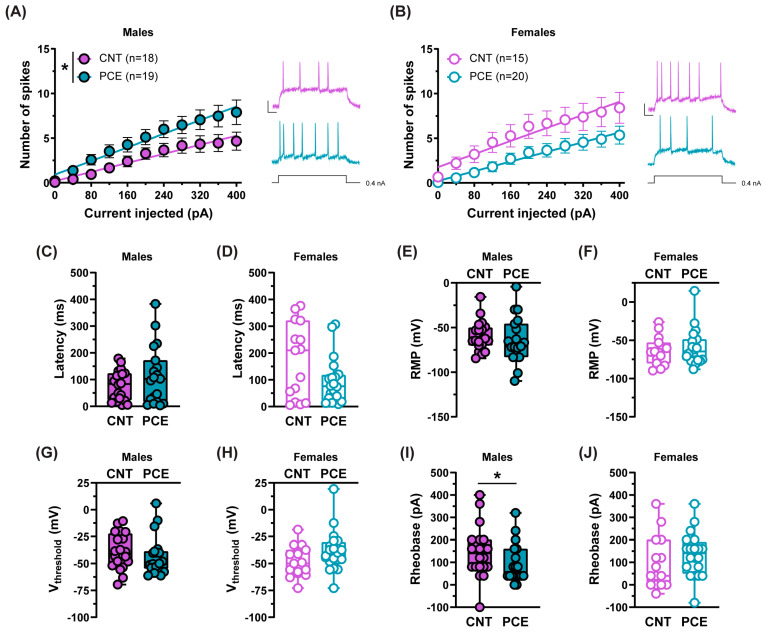
PCE increases the excitability of pyramidal neurons in male offspring at adolescence. (**A**) Male PCE pyramidal neurons (n_rat_ = 10) exhibit increased excitability in response to somatically injected current (* *p* = 0.0442; 2-way RM ANOVA; *p* > 0.05; Holm–Sidak’s) when compared to CNT neurons (n_rat_ = 8). (**B**) No differences were found between female PCE (n_rat_ = 7) and CNT neurons (n_rat_ = 6; *p* = 0.4192; 2-way RM ANOVA; *p* > 0.05; Holm–Sidak’s). Insets show representative traces of evoked action potentials (APs) in response to the maximum current injected; calibration bar: 50 ms, 25 mV. Bar graphs show the intrinsic properties of pyramidal neurons of PCE and CNT offspring: latency for the first evoked AP in males (**C**) (*p* = 0.5639; Mann–Whitney) and females (**D**) (*p* = 0.2173; Mann–Whitney); resting membrane potential (RMP) in males (**E**) (*p* = 0.2924; Mann–Whitney) and females (**F**) (*p* = 0.6747; Mann–Whitney); voltage threshold (V_threshold_) in both sexes ((**G**), *p* = 0.2734 and (**H**), *p* = 0.0674 Mann–Whitney, males and females, respectively); rheobase in males (**I**) (* *p* = 0.0397; Mann–Whitney) and females (**J**) (*p* = 0.1523; Mann–Whitney) (males: CNT n_cells_ = 18, n_ra_t = 8, PCE n_cells_ = 19, n_rat_ = 10; Females: CNT n_cells_ = 15, n_rat_ = 6, PCE nc_ells_ = 20, n_rat_ = 7). Data are represented as average values ± S.E.M.

Since genetic variations in dopamine and *Drd2* modulate PFC excitability [122], our observation of enhanced *Drd2* levels in the PFC of PCE male animals prompted us to investigate the excitability of PFC pyramidal neurons. We focused our analysis on layers V-VI, as neurons in these layers of the mPFC exhibit higher *Drd2* expression [122]. To assess the functional consequences of the observed molecular changes, we used a factorial design (2-way RM ANOVA) that allowed the simultaneous evaluation of both treatment and sex effects on neuronal excitability across different stimulation intensities, and we found that PCE increased mPFC pyramidal cell excitability in male offspring, measured as the greater number of action potentials for the same stimulation intensity in comparison with the CNT rats (2-way RM ANOVA, interaction current × treatment F_(10,350)_ = 1.899, *p* = 0.0442, Holm–Sidak’s *p* > 0.05; Figure 7A). No effect was found in PCE females (2-way RM ANOVA, interaction current × treatment F_(10,330)_ = 1.028, *p* = 0.4192, Holm–Sidak’s *p* > 0.05; Figure 7B).

Since the excitability of pyramidal neurons might depend on their intrinsic properties, we evaluated the latency of the first evoked action potential in response to somatic current injection, but we did not find differences in male offspring (Mann–Whitney, *p* = 0.5639, Figure 7C) or in female progeny (Mann–Whitney, *p* = 0.2173, Figure 7D). The resting membrane potential (RMP) was similar among groups (males: Mann–Whitney, *p* = 0.2924, Figure 7E; females: Mann–Whitney, *p* = 0.6747, Figure 7F), as was the voltage threshold (V_threshold_; males: Mann–Whitney, *p* = 0.2734, Figure 7G; females: Mann–Whitney, *p* = 0.0674, Figure 7H). However, according to an increased excitability, PCE male offspring exhibited a lower rheobase (Mann–Whitney, *p* = 0.0397, Figure 7I). While no differences were found in females (Mann–Whitney, *p* = 0.1523, Figure 7J).

### 3.5. Sex-Specific PCE Effects on Netrin-1 and TH Immunoreactivity in the Medial Prefrontal Cortex and Nucleus Accumbens of Adolescent Rats

**Figure 8:**
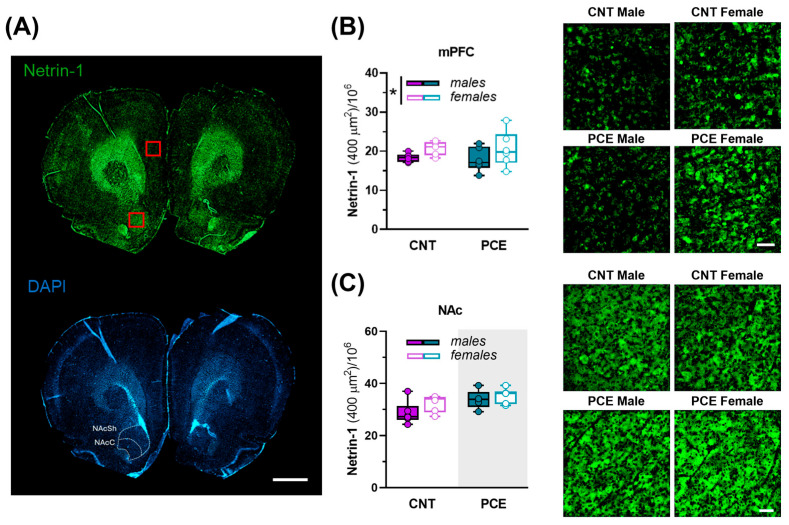
Netrin-1 immunoreactivity in the medial prefrontal cortex (mPFC) and nucleus accumbens (NAc) of PCE and control male and female rats. (**A**) Representative coronal section immunostained for Netrin-1 (top, green) and DAPI (bottom, blu). Red squares indicate representative regions of interest for mPFC and NAc. Scale bar: 1000 µm; (**B**) Graph and related images showing the density of Netrin-1 in the mPFC of control (CNT) and PCE male and female rats (2way ANOVA, * *p* = 0.0428, main effect of sex; n rat = 6–7). Data are reported as mean ± SEM. Scale bar: 50 µm. (**C**) Graph and related images showing the density of Netrin-1 in the NAc of CNT and PCE male and female rats (2way ANOVA, *p* = 0.0148, main effect of PCE (shaded gray area); n rat = 5–6). Scale bar: 50 µm. All data are reported as the mean ± SEM. Abbreviations: NAcC, nucleus accumbens core; NAcSh, nucleus accumbens shell.

The Netrin-1/DCC guidance cue system is involved in the functional organization of dopamine pathways during adolescence [47,123]. Thus, any modification in the expression of Netrin-1 or DCC may dysregulate PFC structure and function in a long-term manner. Therefore, to evaluate the effects of PCE, sex, and their interaction on protein expression in the mPFC and NAc of adolescent rats, we conducted a two-way ANOVA (Figure 8A–C).

In the mPFC, the expression levels of Netrin-1 changed as a function of sex, with male rats showing lower levels than females (2way ANOVA, sex effect, F_(1, 21)_ = 4.648; *p* = 0.0428; PCE effect, F_(1, 21)_ = 0.06430; *p* = 0.8023) (Figure 8A,B). In the NAc, PCE affected Netrin-1 levels, with PCE rats displaying a higher density compared to CNT (2way ANOVA, PCE effect, F_(1, 19)_ = 7.192; *p* = 0.0148; sex effect, F_(1, 19)_ = 3.126; *p* = 0.0931) (Figure 8A,C).

**Figure 9:**
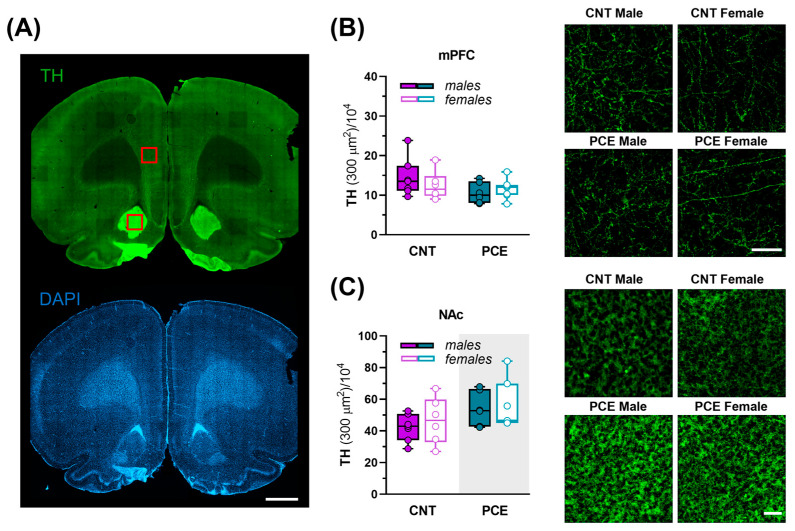
Tyrosine hydroxylase (TH) immunoreactivity in the medial prefrontal cortex (mPFC) and nucleus accumbens (NAc) of PCE and control male and female rats. (**A**) Representative coronal section immunostained for TH (top, green) and DAPI (bottom, blu). Red squares indicate representative regions of interest for mPFC and NAc. Scale bar: 1000 µm; (**B**) graph and related images showing the density of TH in the mPFC of control (CNT) and PCE male and female rats (n_rat_ = 6–7). Data are reported as the mean ± SEM. Scale bar: 100 µm. (**C**) Graph and related images showing the density of TH in the NAc of CNT and PCE male and female rats (2way ANOVA, *p* = 0.0419, main effect of PCE (shaded gray area), n_rat_ = 6–7). Scale bar: 100 µm. All data are reported as mean ± SEM.

To further examine the impact of altered Netrin-1 expression on dopaminergic innervation, we next evaluated the immunoreactivity for tyrosine hydroxylase (TH), the rate-limiting enzyme in dopamine synthesis, in the same brain areas (Figure 9A–C).

In the mPFC, no differences in TH density were found (Figure 9A,B). Conversely, PCE increased TH immunoreactivity in the NAc (2way ANOVA, PCE effect, F_(1, 22) =_ 4.666; *p* = 0.0419; sex effect, F_(1, 22)_ = 0.4173; *p* = 0.5249) (Figure 9A,C) consistently with the upregulation of Netrin-1 in the same brain region.

## 4. Discussion

In the present study, we provide evidence indicating that in rats, PCE elicits transcriptional alterations in the PFC, targeting the dopaminergic system while sparing the endocannabinoid system. PCE rats exhibited increased mRNA expression of both *Drd1* and *Drd2* receptors, with the latter restricted to males. Notably, we found that these PCE-induced transcriptomic changes correlate to epigenetic modifications. Moreover, we demonstrated that these molecular adaptations are associated with heightened pyramidal neuron excitability in the mPFC of male PCE rats alongside a male-specific reduction in Netrin-1-positive fiber density in the same area. Conversely, in the NAc, PCE resulted in a concurrent upregulation of Netrin-1 and TH expression, independent of sex.

A substantial body of literature supports the hypothesis that environmental factors, such as cannabis exposure during critical developmental periods of the PFC, namely gestation and adolescence, are implicated in the etiology of various neuropsychiatric disorders, including SCZ [49,50,51]. The PFC is characterized by a sparse dopaminergic innervation and high expression levels of dopamine receptors [124,125,126]. Dopamine is released at the early stages of mammalian development [57,127], even preceding synaptogenesis. Early receptor activation during this developmental period reshapes brain structure and connectivity, producing lasting anatomical and behavioral changes throughout adulthood [128,129,130,131,132,133,134]. The early appearance and persistence of dopamine during neurodevelopment suggest that an imbalance in dopaminergic signaling may affect the development of brain structures, including the PFC, resulting in abnormal behaviors and brain disorders in later years [100,135]. Of note, as previously suggested [65,66], a hyperdopaminergic state induced by PCE may represent a potential mechanism through which THC could induce psychosis later in life, in line with the dopaminergic hypothesis of SCZ [55]. The findings reported here corroborate our previous data showing the increase in *Drd2* mRNA levels in the PFC of adult male rats exposed to perinatal THC, vulnerable to the development of psychotic-like symptoms [114,115]. This reinforces the idea that pre- and perinatal THC exposure leads to enduring neurobehavioral and molecular changes detectable from early life throughout adulthood. Of note, in rats exposed to prenatal methylazoxymethanol acetate (MAM), a potent DNA methylating agent that disrupts embryonic brain development when administered to pregnant rats [136], a similar upregulation of *Drd2* was observed in the PFC of males [137]. Overall, these data highlight how dopaminergic dysfunction is a hallmark of neurodevelopmental animal models of psychosis [137], which in turn prove to be very useful for modeling various molecular and behavioral components of SCZ. Notably, we show, for the first time to our knowledge, that PCE significantly increases *Drd1* mRNA levels in the PFC. The *Drd1* receptor is implicated in cognitive deficits associated with SCZ, particularly affecting working memory, which relies on proper dorsolateral PFC (DLPFC) functioning. Changes in *Drd1* mRNA levels have been previously documented in the DLPFC of individuals affected by SCZ and mood disorders [138].

Previous studies have explored THC’s impact on epigenetic mechanisms [70], and it has been reported that PCE can lead to epigenetic changes in *Drd2* expression [64,139], thereby disrupting central nervous system development [139]. While earlier work primarily focused on PCE-induced histone modifications [64], our study demonstrates that PCE induces hypomethylation at the regulatory region of the *Drd2* gene, which correlates with the alterations in mRNA levels. These findings align with our previous results observed in adult rats exposed to perinatal THC as well as findings from human SCZ patients [114] and underscore the critical role of DNA methylation during mammalian development [140,141] as well as its association with psychosis risk [142,143,144,145]. Of note, epigenetic modulation of both *Drd1* and *Drd2* is driven by specific miRNAs targeting receptors’ mRNAs. Notably, we found that a reduced expression of miR-17-5p correlates with increased *Drd1* mRNA levels in the PFC of PCE rats. MiRNAs are known to play crucial roles in neurogenesis and brain development [146] as well as being implicated in SCZ pathology [147]. In particular, miR-17-5p and miR-9-5p were investigated here due to their possible role as biomarkers in psychosis. miR-17-5p was found to be dysregulated in the DLPFC of schizophrenic subjects [148] and to be negatively correlated with symptom severity when its plasma levels were analyzed [149]. miR-9-5p is highly expressed in the brain and, consistent with our results, its expression levels were downregulated in the blood of schizophrenic subjects [150]. The changes here observed in these miRNAs levels may also be interesting in light of a recent study highlighting how PCE alters miRNAs expression profiles in rhesus macaques [96]. Moreover, we observed a correlation between miR-9-5p expression levels and DNA methylation levels at the *Drd2* locus within the PFC of PCE rats. This supports the hypothesis that there is an intricately linked relationship between DNA methylation and miRNAs in governing gene expression [151]. Rather than functioning as isolated processes, these epigenetic mechanisms may operate as collaborative components of a unified regulatory network, forming a dynamic, interconnected system. This multifaceted machinery adaptively fine-tunes gene activity through different molecular strategies, underscoring its critical role in cellular function and disease pathogenesis. In this context, according to Moreno et al. [139], we could hypothesize that PCE might function as an epigenetic factor by inducing lasting molecular changes that affect gene regulation critical for neurodevelopment. Epigenetic mechanisms represent a pivotal interface between environmental influences, such as cannabinoid exposure, and genomic activity, creating a molecular signature of the exposure that can influence neural development throughout the lifespan. In fact, disruptions of the epigenome are widely believed to drive the lasting, often tissue-specific changes in gene expression and behavior caused by cannabinoids [85,86]. These alterations constitute a form of the “molecular trace” of developmental exposure, potentially explaining how time-limited cannabis exposure during prenatal development can result in the persistent neurobiological changes observed in adolescence. Importantly, such epigenetic reprogramming is not only likely to influence the directly exposed individual but might be transmitted through subsequent generations, suggesting a possible transgenerational process for susceptibility to neuropsychiatric disease following developmental cannabis exposure.

In agreement with Bara and colleagues [111], we did not observe any change in ECS gene expression in the PFC of PCE rats. ECS plays a crucial role in shaping lifespan neurodevelopment [152], and THC exposure may affect the temporal and spatial control of ECS signaling at critical stages of neuronal development, such as the prenatal one [35]. However, the lack of alterations in the ECS system might either reflect a compensatory mechanism, triggered by the developing brain to counteract exogenous cannabinoid exposure (i.e., receptor desensitization, endocannabinoid production adjustments, allosteric modulation), or a form of resilience of the ECS signaling pathways within the PFC thanks to its role in synaptic plasticity. 

It is important to note that our statistical analyses revealed interesting patterns that warrant careful interpretation. In some cases, we observed significant PCE effects in the combined sample without significant effects in either sex when analyzed separately. This pattern suggests that these effects may be present in both sexes but with insufficient power to be detected when the sample is split by sex. In other instances, such as with *Drd2* expression, we found effects in the combined sample that were only present in males when analyzed by sex. While the PCE effect on *Drd2* mRNA levels could only be detected in males, this does not definitively rule out effects in females, which may be smaller. These patterns highlight the complexity of interpreting sex-dependent effects in response to PCE.

Our electrophysiological analysis revealed an increased excitability of pyramidal neurons in male adolescent PCE offspring, consistent with previous findings in adult PCE male progeny [111], thus suggesting that PCE-induced alterations in PFC neuron excitability are already manifest at adolescence and persist until adulthood. Since PFC layers V-VI pyramidal neurons are primarily projecting cells [153], one might speculate that PCE male offspring manifest stronger connectivity between PFC neurons and downstream targets. Our observations support the evidence that synaptic inputs might increase PFC neuron excitability through a *Drd2*-dependent mechanism [122,154]. Although *Drd2* activation is classically associated with decreased neuronal excitability, it has also been shown that it can elicit an afterdepolarization that enhances layer V pyramidal neuron excitability and increases outputs to subcortical structures [122,154]. Conversely, a decreased excitability has been detected in *Drd2* knockout PFC neurons [155]. Therefore, the observation of an elevated *Drd2* expression in the PFC of adolescent male PCE progeny may partially contribute to the enhanced excitability of PFC pyramidal neurons. Additionally, PCE impairs long-term potentiation (LTP) within brain regions critical for learning and memory, such as the hippocampus [110,156,157]. Considering that the hippocampus typically refines and filters information before sending it to the PFC, one can speculate that the reduced hippocampal LTP could lead to a less precise flow of information toward the PFC. As a result, the PFC would have to process a less refined signal [158]. We further presume that this altered information processing may contribute to the increased excitability of PFC pyramidal neurons, potentially representing a maladaptive or compensatory mechanism to maintain cognitive functions, which are disrupted by PCE [110,156,157,159]. While our study focuses on the excitability of PFC neurons as a potential correlate for altered D2 receptor expression, future research is essential to unravel the complex neurodevelopmental consequences of PCE and their relations with altered afferent input processing.

Our findings provide the first evidence indicating that PCE significantly impacts the Netrin-1/DCC pathway, consistent with previous reports demonstrating that drug abuse alters dopamine system maturation [47,160]. With the Netrin-1/DCC signaling pathway being a well-established axon guidance signal which, during adolescence, enables the correct anatomical and functional establishment of the mesocorticolimbic dopamine circuitry [103], our findings highlight how disruptors of the ECS interfere with dopamine system neurodevelopment. As a chemoattractant, Netrin-1 guides dopamine axons originating in the VTA to their targets in the PFC and NAc. This process, crucial for establishing appropriate synaptic connections, is vital for the proper development of executive functions, emotional regulation, and reward processing. Since Netrin-1 levels in the NAc as well as DCC expression in the VTA decrease during development [160], our findings that PCE upregulates this signaling in the NAc, along with increasing TH expression levels in this area, at adolescence suggest a potential deregulation in the maturation of the mesocorticolimbic dopamine system. During adolescence, mesolimbic dopamine axons establish local connections within the NAc, while mesocortical dopamine neurons continue axonal growth toward the PFC [161]. The NAc serves as a central control point determining whether dopamine axons arising from the VTA will terminate and constitute the mesolimbic system or extend toward the PFC, thus becoming part of the mesocortical system [123,162,163,164]. Since this mechanism is modulated by Netrin-1/DCC levels within the NAc, and disruptions of these levels can impair PFC connectivity and function [102,123,165], PCE-induced upregulation of Netrin-1 signaling in the NAc may suggest an impairment in the functional organization of mPFC. Notably, in the mPFC, Netrin-1 expression exhibits sexual dimorphism, with upregulated Netrin-1 levels observed in females. While the functional implications of this sexual dimorphism remain unclear, this sex-specific upregulation may reflect a protective mechanism to contrast the deleterious effects of PCE on the dopaminergic system. This hypothesis is consistent with prior evidence showing sex-specific changes in Netrin-1 expression in adolescent female rats following amphetamine to contrast alterations in dopaminergic connectivity and cognitive outcomes [160]. Conversely, the observed increased TH expression in the NAc of PCE adolescent rats is consistent with previous findings indicating that PCE alters brain reward circuits, leading to hyper-responsiveness to reward-related cues [60]. Specifically, PCE upregulated dopamine release responses to food- and drug-predictive cues, induced an overrepresentation of effort-encoding NAc neural dynamics, and enhanced impulsivity [60]. Hence, PCE-induced alterations in Netrin-1 and TH expression in the NAc provide potential underpinnings of this hyperdopaminergia, although multiple mechanisms may contribute to this phenomenon.

In summary, these findings highlight alterations of the PFC dopaminergic system as a function of PCE, while maintaining stable ECS gene expression, suggesting a complex interplay between these systems during important neurodevelopmental periods. Moreover, our data bolster the hypothesis that maternal cannabis consumption can lead to neurodevelopmental alterations that may predispose individuals to psychosis later in life through epigenetic mechanisms [166,167].

Further studies will be required to clarify if PCE triggers other behavioral dysfunctions, as previously investigated [65], by altering the mesocorticolimbic dopamine circuitry to assess early signs of altered neurodevelopmental trajectories related to PCE-related endophenotypes.

## Figures and Tables

**Table 1:** List of primers used for quantitative real-time polymerase chain reaction (qRT-PCR).

Gene		*5′-Forward Primer-3′*	*5′-Reverse Primer-3′*
Housekeeping	*ß-Actin*	AGATCAAGATCATTGCTCCTCCT	ACGCAGCTCAGTAACAGTCC
	*Gapdh*	AGACAGCCGCATCTTCTTGT	CTTGCCGTGGGTAGAGTCAT
	*18S*	ACGGACCAGAGCGAAAGCAT	TGTCAATCCTGTCCGTGTCC
Endocannabinoid System	*Cnr1*	TTCCACCGTAAAGACAGCCC	TCCACATCAGGCAAAAGGCC
	*Cnr2*	TTGACCGATACCTATGTCTGTGC	TGCTTTCCAGAGGACATACCC
	*Trpv1*	ATTGAACGGCGGAACATGACG	ATCTCTTCCAGCTTCAGCG
	*Nape-pld*	TGTCCCGGGTTCCAAAGAGGAGC	ACCATCAGCGTCGCGTGTCC
	*Dagl-α*	ATTCTCTCCTTCCTCCTGC	ATTTGGGCTTGGTGCTTCG
	*Faah*	ATGGAAGTCCTCCAAGAGC	TAGAGCTTTCAGGCATAGCG
	*Magl*	ACGTGAACACCGTCCAGAAG	TTGGCAGCAAGGACCTTCAA
Dopaminergic System	*Drd1*	TCGAACTGTATGGTGCCCTT	AAGAATTCGCCCACCCAAAC
	*Drd2*	TACGTGCCCTTCATCGTCAC	GTGGGTACAGTTGCCCTTGA
	*Drd3*	ATTCGGCAGTTTTCAATAAGG	GGGTGTCTCAAGGCAGTGTC
	*Dat*	AGCTACCATGCCCTATGTGG	ATCAGCACTCCAAACCCAAC

**Table 2:** Details of sequences and primers employed for the DNA methylation analysis by pyrosequencing. Rn = Rat; CpG = C-phosphate-G; F = forward primer; Biot_R = biotinylated reverse primer; S = sequencing primer. Bold text = CpG sites analyzed.

*Gene*	*Sequence*	*n° CpG Sites*	*5′-Primers-3′*
***Rn_Drd1***	gg**cg**tggg**cg**tggggagggt**cg**gctctgat tc**cg**agctttgggtggaacttgaggttgg	4	**F:** GGGTAGTGTTTTGGGTTAGT **Biot_R:** TCTCTTCAAACCAACCTCAAAT **S**: AGTGTTTTGGGTTAGTAG
***Rn_Drd2***	ttcc**cg**a**cg**cc**cg**agg**cg**caatctgcc**cg**t**cg**ga	6	Included in the Qiagen Assay PM00586096

**Table 3:** Gene expression of ECS elements (receptors and metabolic enzymes) in the PFC of prenatal cannabis-exposed (PCE) rats. Data are reported as 2^−ΔΔCt^ values calculated by the Delta–Delta Ct (ΔΔCt) method versus control (CNT) rats. Expression was normalized to GAPDH, β-actin, and 18S. Data are reported as the mean ± SEM (n = 11–15 rats/group). *p*-values are depicted.

ECS Genes	CNT (Mean ± SEM)	PCE (Mean ± SEM)	*p*-Values
*Cnr1*	1.34 ± 0.29	1.10 ± 0.27	0.4777
*Cnr2*	1.09 ± 0.12	1.61 ± 0.28	0.2456
*Trpv1*	1.11 ± 0.14	1.30 ± 0.12	0.3369
*Nape-pld*	1.16 ± 0.17	1.19 ± 0.25	0.7068
*Dagl-α*	1.13 ± 0.13	1.06 ± 0.17	0.6397
*Faah*	1.03 ± 0.08	1.08 ± 0.14	>0.9999
*Magl*	1.20 ± 0.20	1.14 ± 0.24	0.7291

## Data Availability

Data can be provided from the corresponding authors upon reasonable request.

## Supplementary Materials

The following supporting information can be downloaded at: https://www.mdpi.com/article/10.3390/cells14120904/s1, Table S1: Gene expression of ECS elements (receptors and metabolic enzymes) in PFC of prenatal Cannabis exposed (PCE) rats, stratified according to sex. Figure S1. Schematic representation of rat (A) Drd1 (Transcript Drd1-202, ENSRNOT00000117894.1, genome assembly mRatBN7.2) and (B) Drd2 (Transcript Drd2-202, ENSRNOT00000083419.2, genome assembly mRatBN7.2) genes. Figure S2. miR-30b-5p expression levels of all the study samples.

## Abbreviations

The following abbreviations are used in this manuscript:

18S	18s ribosomal RNA
2-AG	2-arachidonoylglycerol
β-ACT	Beta Actin
aCSF	Artificial cerebrospinal fluid
AEA	Anandamide
Cnr1	Cannabinoid Receptor Type1
Cnr2	Cannabinoid receptor Type2
Dagl-α	Diacylglycerol Lipase Alpha
Dat	Dopamine Transporter
Dcc	Deleted in Colorectal Cancer
DLPFC	Dorsolateral Prefrontal Cortex
Drd1	Dopamine D1 receptor
Drd2	Dopamine D2 receptor
Drd3	Dopamine D3 receptor
ECS	Endocannabinoid system
Faah	Fatty Acid Amide Hydrolase
Gapdh	Glyceraldehyde-3-phosphate dehydrogenase
GD	Gestational day
Magl	Monoglyceride Lipase
MAM	Methylazoxymethanol acetate
mPFC	Medial prefrontal cortex
NAc	Nucleus accumbens
NAcC	Nucleus accumbens core
NacSh	Nucleus accumbens shell
Nape-Pld	N-Acylphosphatidylethanolamide-Phospholipase D
PCE	Prenatal cannabis exposure
PND	Postnatal day
PFC	Prefrontal cortex
SCZ	Schizophrenia
TH	Tyrosine hydroxylase
THC	Delta-9-tetrahydrocannabinol
Trpv1	Transient Receptor Potential Cation Channel Subfamily V Member 1
