# Supplementary material for: Prenatal Delta-9-Tetrahydrocannabinol Exposure Induces Transcriptional Alterations in Dopaminergic System with Associated Electrophysiological Dysregulation in the Prefrontal Cortex of Adolescent Rats
Source: Cells. 2025 Jun 14;14(12):904. doi: 10.3390/cells14120904 (PMC12191062; doi:10.3390/cells14120904)
Supplement: Supplementary file 1 [file cells-14-00904-s001.zip › cells-3608349-supplementary.pdf]

cells

MDPI

Article

Prenatal Delta-9-tetrahydrocannabinol exposure induces transcriptional alterations in dopaminergic system with associated electrophysiological dysregulation in the prefrontal cortex of adolescent rats

Supplementary material

Martina Di Bartolomeo[1,†], Sonia Aroni[2,†], Marcello Serra[2], Valeria Serra[2], Francesca Martella[1], Federica Gilardini[1], Miriam Melis [2,*,‡] and Claudio D'Addario [1,3,*,‡]

[1] Department of Bioscience and Technology for Food, Agriculture and Environment, University of Teramo, Teramo, Italy.
[2] Department of Biomedical Sciences, Division of Neuroscience and Clinical Pharmacology, University of Cagliari, Cagliari, Italy.
[3] Department of Clinical Neuroscience, Karolinska Institutet, Stockholm, Sweden
* Correspondence: myriam@unica.it (M.M.); cdaddario@unite.it (C.D.); +39-070-675-4322/4340 (M.M.); +39-0861-266877 (C.D.)
† These authors contributed equally to this work
‡ Co-senior Authors

Academic Editor: Firstname Lastname

Received: date
Revised: date
Accepted: date
Published: date

Citation: To be added by editorial staff during production.

Copyright: © 2025 by the authors. Submitted for possible open access publication under the terms and conditions of the Creative Commons Attribution (CC BY) license (https://creativecommons.org/licenses/by/4.0/).

SUPPLEMENTARY TABLES

Table S1 Gene expression of ECS elements (receptors and metabolic enzymes) in PFC of prenatal Cannabis exposed (PCE) rats, stratified according to sex.

	**MALES**			**FEMALES**		
**ECS genes**	**CNT (mean ± SEM)**	**PCE (mean ± SEM)**	**P values**	**CNT (mean ± SEM)**	**PCE (mean ± SEM)**	**P values**
Cnr1	1.32 ± 0.42	1.05 ± 0.33	0.8290	1.36 ± 0.43	1.15 ± 0.44	0.6457
Cnr2	1.07 ± 0.15	1.42 ± 0.30	0.5338	1.07 ± 0.14	1.82 ± 0.55	0.2844
Trpv1	1.09 ± 0.19	1.11 ± 0.17	0.9740	1.12 ± 0.21	1.48 ± 0.18	0.2378
Nape-pld	1.14 ± 0.23	1.48 ± 0.39	0.8048	1.16 ± 0.26	0.84 ± 0.17	0.2928
Dagl-α	1.19 ± 0.22	0.99 ± 0.30	0.6389	1.06 ± 0.15	1.13 ± 0.22	> 0.9999
Faah	1.05 ± 0.15	1.23 ± 0.23	0.7460	1.02 ± 0.09	0.96 ± 0.18	0.5887
Magl	1.23 ± 0.35	1.30 ± 0.37	> 0.9999	1.15 ± 0.23	0.91 ± 0.27	0.5093

Data are reported as $2^{-\Delta\Delta Ct}$ values calculated by Delta–Delta Ct (ΔΔCt) method versus control (CNT) rats. Ex-pression was normalized to GAPDH, β-actin and 18S. Data are reported as mean ± SEM (n=5–7 rats/group, male rats; n=5/8 rats/group, female rats). P values are depicted.

SUPPLEMENTARY FIGURES

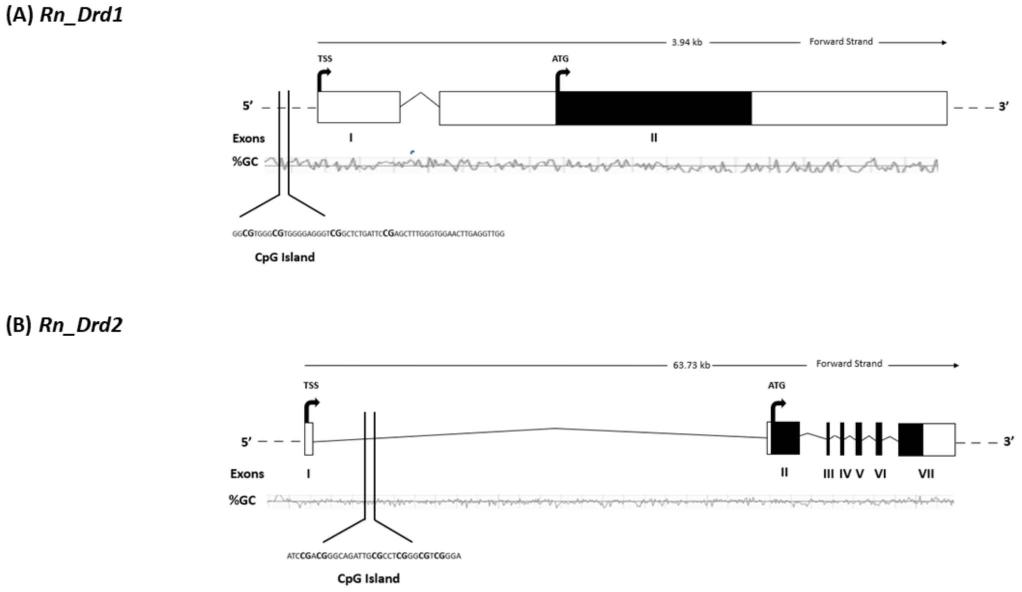

Supplementary Figure S1. Schematic representation of rat (A) *Drd1* (Transcript Drd1-202, ENSRNOT00000117894.1, genome assembly mRatBN7.2) and (B) *Drd2* (Transcript Drd2-202, ENSRNOT00000083419.2, genome assembly mRatBN7.2) genes. ATG is the translation start site. Shown are the location of CpG islands, exons, introns and TSS (transcription start site). Coding regions of exons are shown darker. Sequence of CpG islands is depicted.

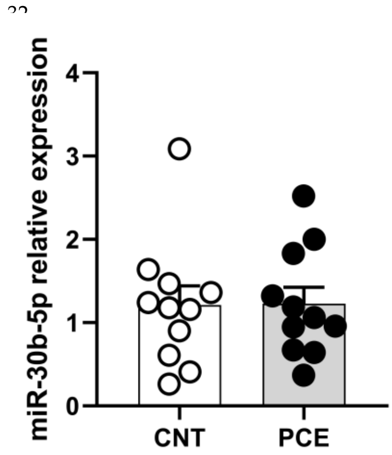

Supplementary Figure S2. miR-30b-5p expression levels of all the study samples. miRNA expression data are reported as $2^{-\Delta\Delta Ct}$ values calculated by Delta–Delta Ct (ΔΔCt) method versus CNT. Expression was normalized to U6. Data are reported as mean ± SEM (n=11–11 rats/group)
